# PILRα binds an unknown receptor expressed primarily on CD56^bright^ and decidual-NK cells and activates NK cell functions

**DOI:** 10.18632/oncotarget.8397

**Published:** 2016-03-27

**Authors:** Yael Ophir, Alexandra Duev-Cohen, Rachel Yamin, Pini Tsukerman, Yoav Bauman, Moriya Gamliel, Ofer Mandelboim

**Affiliations:** ^1^ The Lautenberg Center for General and Tumor Immunology, The BioMedical Research Institute Israel-Canada of The Faculty of Medicine, The Hebrew University Hadassah Medical School, Jerusalem, Israel

**Keywords:** NK, PILRa, CD56 bright, Immunology and Microbiology Section, Immune response, Immunity

## Abstract

Natural Killer (NK) cells are innate immune lymphocytes specializing in recognition and killing of tumors and pathogens, using an array of activating and inhibitory receptors. NK inhibition is mediated by a large repertoire of inhibitory receptors, whereas a limited number of activating NK cell receptors execute NK cell activation. The ligands recognized by the activating receptors are stress-induced, pathogen derived, tumor specific and even self ligands. However, the full spectrum of NK cell receptors and ligands that control NK cell activity remains uncharacterized. Here we demonstrate that Paired Ig-Like type 2 Receptor Alpha (PILRα), binds a distinct human NK cell sub-population present in the peripheral blood and also in the decidua. We further demonstrate that the interaction of NK cells with PILRα expressing targets lead to elevated IFNγ secretion and cytotoxicity. In conclusion, we present here a novel NK activating ligand which binds and activates an unknown NK receptor expressed on a unique NK cell subset.

## INTRODUCTION

NK cells express a vast combinatorial array of receptors that can activate or inhibit NK-mediated cytotoxicity and cytokine secretion, in response to oncogenic transformation, bacterial, fungal and viral infections [1][2]. When encountered with target cells, the decision to kill or spare the target cells is determined by a balance of signals delivered by these receptors, which recognize a variety of ligands on the target cells [3][4].

NK cell cytotoxicity is positively controlled by activating receptors. The most prominent family of NK cell activating receptors is the Natural Cytotoxicity Receptors (NCR), which includes: NKp30 [5], NKp44 [6], and NKp46 [7]. While NKp46 and NKp30 are constitutively expressed by all NK cells, NKp44 is only expressed following activation [8]. The NCRs recognize a variety of tumor and pathogen-derived molecules. For instance, NKp30 recognizes B7H6 which is specifically expressed on tumor cells [9] and the pp65 protein of human cytomegalovirus [10]. NKp44 binds the E-protein of Dengue and West Nile viruses [11]. NKp46 directly recognizes the *Fusobacterium nucleatum* [12] and *Mycobacterium tuberculosis* [13] bacteria via unknown ligands. However the full repertoire of NCR ligands, including self and tumor ligands, remains to be established.

The most characterized and the first NCR ligands discovered were the influenza virus hemagglutinin (HA) and the Sendai virus HA-neuraminidase, which bind both NKp44 and NKp46 [14][15]. The receptor-ligand binding characteristics of NKp46 to HA was previously established as O-linked glycosylation dependent, specifically relying on the sugar-carrying residue Thr 225 on NKp46 [16]. Furthermore, sialylated residues were also demonstrated to be involved in the interaction of NKp46 with its unknown tumor ligand [16], suggesting that sialylated residues dictate the broad spectrum of virally-infected and tumor cells recognized by NKp46. The identity of the cellular proteins that interact with NKp46 in a sialic acid-dependent manner remains unknown.

Paired Ig-Like type 2 Receptor alpha (PILRα) was previously shown to recognize O-glycosylated mucin receptors such as PILR-associating neural protein (PANP), neuronal differentiation and proliferation factor-1 (NPDC1) and collectin-12 (COLEC12) [17][18]. PILRα is a type I transmembrane receptor, expressed primarily on cells of the myelomonocytic lineage, including granulocytes, monocytes, macrophages and dendritic cells [19][20]. Here we show that PILRα binds to a subset of human NK cells and that this binding leads to increased NK mediated IFNγ secretion and killing.

## RESULTS

### PILRα-Ig binds an unknown receptor, expressed on a specific subset of human NK cells

**Figure 1:**
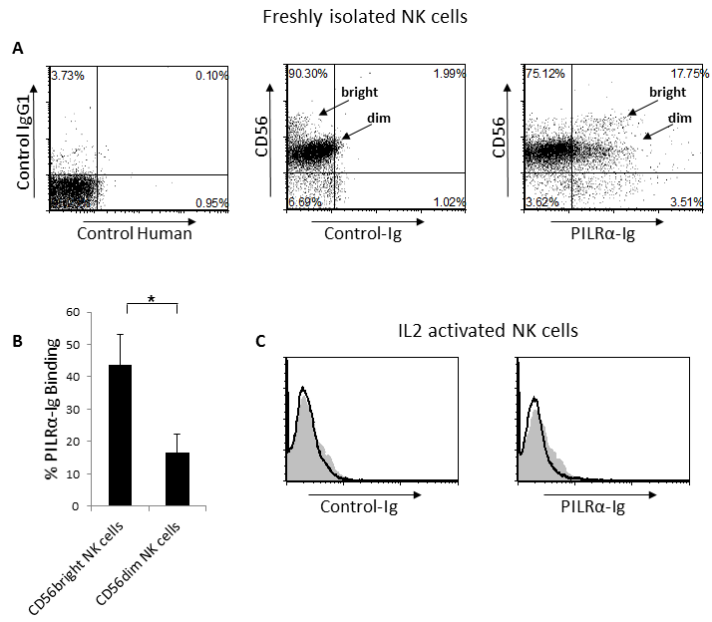
PILRα-Ig binds an unknown receptor on NK cells **A.** Dot plot FACS staining of freshly isolated NK cells, left is the setup controls, middle is the double staining with anti-CD56 and with control-Ig fusion protein and right is the double staining of PILRα-Ig and CD56. The CD56dim and CD56bright NK cells are indicated by an arrow in the middle and right dot blots. **B.** Quantification of the percentages of PILRα-Ig binding to the different NK cells populations. Figure summarizes 7 independent staining. *p < 0.05, NS-not significant. Statistics was performed using student *T*-test. **C.** FACS staining of IL2 activated NK cells. Grey filled histograms are background control. Black line histograms represent specific staining, as indicated. Figure show one representative experiment out of 5 performed (in A and C).

We have previously shown that the viral HA protein binds NKp44 and NKp46, consequently leading to an increase in NK cell mediated killing of influenza-infected cells [14][15]. We further demonstrated that HA interacts with NKp46 in a sialic acid dependent manner, specifically via the O-linked glycosylated Thr 225 [16]. Because, PILRα was shown to bind O-linked glycosylated receptors, such as Collectin12, PANP and NPDC [17][18], we sought to investigate whether PILRα might also interact with NKp46 and NKp44. To test this, we initially generated a PILRα-Ig fusion protein composed of the extracellular part of PILRα fused in frame with human IgG1 (named PILRα-Ig). The fusion protein was produced in 293T cells and purified on protein G columns. We then used PILRα-Ig in FACS assays to assess binding to freshly isolated NK cells. PILRα-Ig showed binding to a portion of the NK cells, comprised of both CD56^dim^ and CD56^bright^ NK cell sub-populations (Figure 1A). Quantification of the percentage of PILRα-Ig binding to the different sub-populations, using various donors, reveals that PILRα-Ig binds approximately 50% of the CD56^bright^ cells and 15% of the CD56^dim^ cells (Figure 1B). Interestingly, while we observed PILRα-Ig binding to freshly isolated NK cells, PILRα-Ig showed no binding to IL2 activated NK cells (Figure 1C).

### PILRα does not interact with NKp44 or NKp46

**Figure 2:**
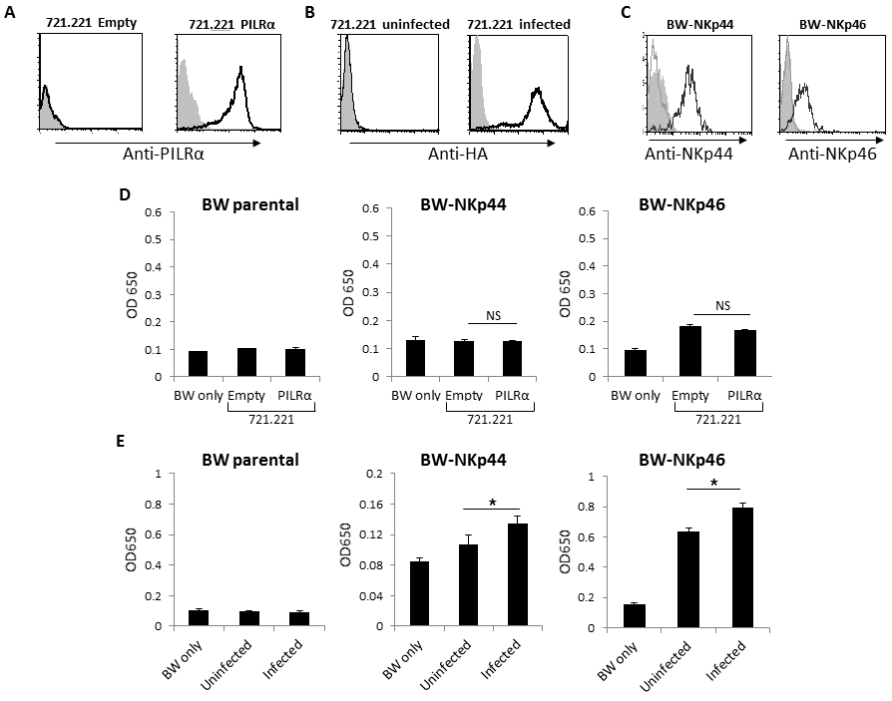
PILRα expressing cells does not increase IL2 secretion of BW-NKp44 and BW-NKp46 cells **A.** FACS staining of 721.221 cells transfected either with an empty vector as control, or with PILRα. Grey filled histograms are background control staining. Black line histograms represent specific anti-PILRα staining. **B.** FACS staining of 721.221 cells in the presence or absence of PR8 Influenza. Grey filled histograms are background control staining. Black line histograms represent specific anti-HA staining. **C.** FACS staining of parental or NKp44 and NKp46 transfected BW cells. Grey filled histograms are background control staining. Grey line histograms represent the staining of the parental BW cells with the appropriate mAbs, black line histograms represent staining of NKp44/46 transfected BW cells with the appropriate mAbs. **D.** IL2 secretion from parental BW (left), BW-NKp44 (middle) and BW-NKp46 (right) cells. IL2 secretion was measured by ELISA (OD 650nm) following incubation with 721.221 cells transfected either with an empty vector or with PILRα. **E.** IL2 secretion of parental BW (left), BW-NKp44 (middle) and BW-NKp46 (right) cells. IL2 secretion was measured by OD 650nm following incubation with 721.221 in the presence (designated infected) or in the absence (designated uninfected) of influenza PR8. Figure show combine 3 independent experiments. *p < 0.05, NS-not significant. Statistics was performed using student *T*-test.

The NCRs expression pattern on NK cells is well-characterized: NKp44 expression is induced following activation, while it is almost completely absent on fresh NK cells [8]. NKp46, on the other hand, is expressed on both fresh and IL2 activated NK cells [8]. Thus, it seems as if PILRα does not interact with NKp46 or NKp44 since not all freshly isolated NK cells were stained by PILRα-Ig (Figure 1A) and IL2 activated NK cells expressing NKp44 and NKp46 (data not shown) were not recognized at all by PILRα-Ig (Figure 1C). Nevertheless, to further demonstrate that PILRα does not interact with NKp44 or NKp46, we prepared 721.221 cells expressing a control empty vector and 721.221 cells expressing PILRα (Figure 2A). We also incubated 721.221 cells with PR8 influenza to be used as a positive control (Figure 2B).

To test whether PILRα can bind and activate NKp44 or NKp46, we used a reporter cell based assay. We expressed in the BW thymoma cell line chimeric proteins composed of the extracellular portions of NKp46 and NKp44, fused to the mouse ζ-chain (Figure 2C). Upon engagement of the fused chimeric NCRs with their ligand, the BW cells secrete IL2, thus reporting the binding and the functional characteristic of the tested interaction. We incubated the various BW cells with the different targets and determined the amount of IL2 in the culture supernatants by ELISA. No increased secretion of IL2 was observed when the parental BW and BW-NKp44 were incubated with 721.221 cells expressing an empty vector or PILRα (Figure 2D). This indicates that 721.221 cells do not express ligands for NKp44 and that NKp44 does not interact with PILRα. Increased IL2 secretion was observed when BW-NKp46 cells were incubated either with the 721.221 empty vector cells or with PILRα expressing 721.221 cells (Figure 2D), indicating that 721.221 cells express an unknown ligand for NKp46. However, since no difference in IL2 secretion was detected when BW-NKp46 cells were incubated with 721.221 cells or 721.221 cell expressing PILRα (Figure 2D), we concluded that NKp46 do not interact with PILRα. We previously reported that both NKp44 and NKp46 interact with viral HA and this interaction activates the BW-NKp44 and BW-NKp46 reporter systems [21][22]. Indeed, a significant increase in IL2 levels was observed in the culture supernatant of BW-NKp44 and BW-NKp46 cells following incubation with PR8 influenza 721.221 cells (Figure 2E), indicating that both the BW-NKp44 and BW-NKp46 reporter systems function properly. These combined results further indicate that PILRα does not bind NKp44 nor NKp46.

### PILRα-Ig positive CD56^bright^ NK clones are activated to secrete IFNγ following PILRα engagement

**Figure 3:**
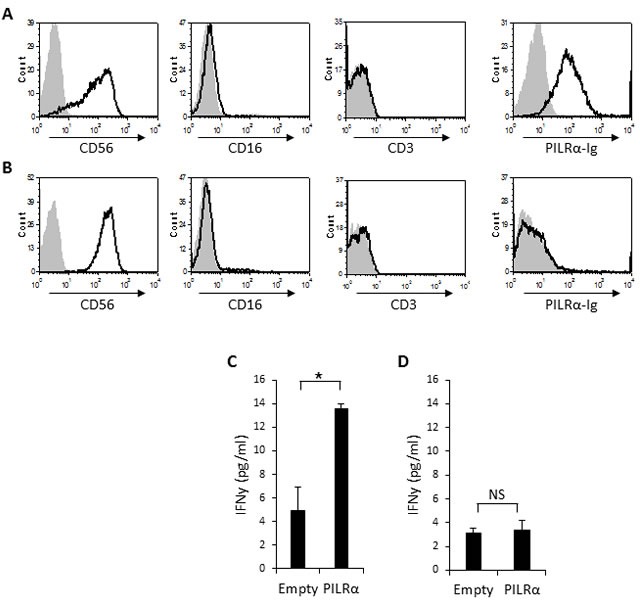
PILRα-Ig binds some CD56 bright NK clones and causes IFNγ secretion **A.**, **B.** FACS staining of isolated CD56^bright^, CD16^negative^, CD3^negative^ NK clones. Grey filled histograms are background control staining. Black line histograms represent specific staining, as indicated. (A) PILRα-Ig positive NK clone. (B) PILRα-Ig negative clone. **C.**, **D.** IFNγ secretion by PILRα-Ig positive NK clones (C) and by PILRα-Ig negative NK clones (D) following incubation with 721.221 cells transfected either with an empty vector as control, or with PILRα. Figure combine 3 independent experiments. *p < 0.05, NS-not significant. Statistics was performed using student T-test.

Two distinctive sub populations of NK cells are present in peripheral blood naïve NK cells. The majority (approximately 90%) of the naïve human NK cells express CD56 at intermediate levels (CD56^Dim^), whereas a minor population of the naive NK cells (approximately 10%) expresses CD56 at high levels (CD56^Bright^) [23–25]. To test the functionality of the PILRα-Ig-positive NK cells we isolated NK clones from the CD56^bright^CD16^Negative^ sub-population. We isolated CD56^bright^ clones, since around 50% of the population bound PILRα-Ig (Figure 1B). Two types of clones, both negative for CD3 (Figure 3A and 3B) were isolated: clones which were bound to PILRα-Ig (example is shown Figure 3A) and clones that did not (example is shown in Figure 3B).

We then performed IFNγ secretion assays. 721.221 cells expressing an empty vector or PILRα, were incubated with PILRα-Ig positive or negative NK clones, and IFNγ levels were measured in the culture supernatants. Importantly, a significant increase in IFNγ secretion was observed when PILRα-Ig positive NK clones were incubated with the PILRα expressing 721.221 cells, compared to the empty vector control (Figure 3C). The effect was specific and was restricted to NK clones that were stained with PILRα-Ig, as these effects were not observed in the PILRα-Ig negative NK clones (Figure 3D). Similar results were obtained with additional NK clones (data not shown).

### PILRα-Ig positive NK clones show increased cytotoxicity and degranulation upon interaction with PILRα expressing target cells

**Figure 4:**
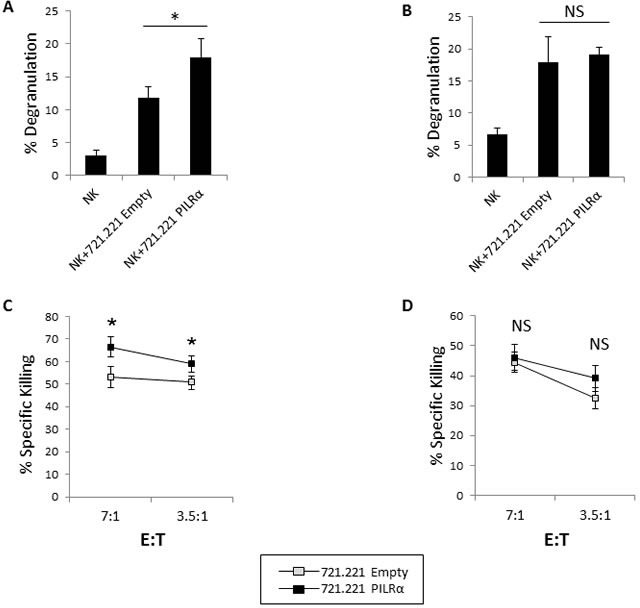
PILRα-Ig positive clones show increased cytotoxicity and degranulation upon interaction with PILRα expressing target cells **A.**, **B.** Percent of NK cell degranulation by PILRα-Ig positive NK clones (A) and by PILRα-Ig negative NK clones (B) following incubation with 721.221 cells transfected either with an empty vector as control, or with PILRα. Degranulation percentage was determined by CD107A FACS staining, *p < 0.05. **C.**, **D.** Percent of specific NK cell killing by PILRα-Ig positive NK clones (C) and by PILRα-Ig negative NK clones (D) following incubation with 721.221 cells transfected either with an empty vector as control, or with PILRα. Figure show one representative experiment out of at least 3 performed. *p < 0.05, NS-not significant. Statistics was performed using student *T*-test.

To further examine the functional significance of PILRα-Ig positive NK cell clones, we tested the degranulation and killing potential of these NK clones. In the degranulation assays, 721.221 cells expressing an empty vector or PILRα, were incubated with PILRα-Ig positive or negative NK clones. Notably, significant degranulation was observed when PILRα-Ig positive NK clones were incubated with the PILRα expressing 721.221 cells, compared to the empty vector control (Figure 4A), while no significant increase in degranulation was observed in the PILRα-Ig negative NK clones (Figure 4B). Similar results were obtained with additional NK clones (data not shown). NK cell degranulation strongly correlated with the killing potential of the NK clones, however, it do not directly measure NK cell cytotoxicity. We therefore also conducted NK cell cytotoxicity assays. PILRα-Ig positive and negative NK clones were incubated with the 721.221 targets and the percent of specific NK mediated killing was measured. PILRα-Ig positive NK clones were consistently more potent in eliminating PILRα expressing targets than non-expressing PILRα targets (Figure 4C). The effect was specific and PILRα-Ig positive restricted as these effects were not observed in the PILRα-Ig negative NK clones (Figure 4D). Similar results were obtained with additional NK clones (data not shown). These results indicated that PILRα expressing targets not only lead to elevated IFNγ secretion, but also induced increased cytotoxicity and degranulation of NK cells.

### Increased IFNγ secretion and killing by PILRα-Ig positive decidual NK in response to PILRα expressing target cells

**Figure 5:**
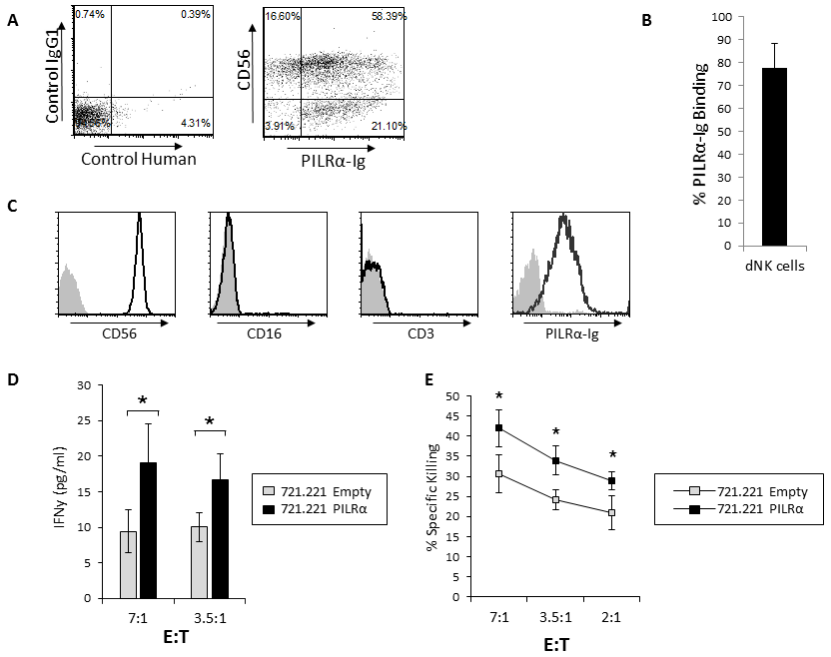
Increased IFNγ secretion and killing of PILRα-Ig positive dNK in response to PILRα expressing target cells **A.** FACS dot blot staining of dNK cells with control-Ig (left) and with anti CD56 and PILRα-Ig (right). **B.** Quantifying the percentage of PILRα-Ig binding to dNK Cells. Figure summarizes 6 independent staining. **C.** FACS staining of dNK PILRα-Ig positive clone with anti CD56, CD16, CD3 and PILRα-Ig. **D.** IFNγ secretion from PILRα-Ig positive dNK cells, following incubation with 721.221 cells transfected either with an empty vector as control, or with PILRα, at two effector to target ratios as indicated, *p < 0.05. **E.** Percent specific killing of the empty vector or PILRα transfected 721.221 target cells by PILRα positive dNK cells at various E:T ratios, as indicated. Figure show one representative experiment out of 3 performed. *p < 0.05, Statistics was performed using student *T*-test.

The CD56^bright^ NK cell population is the predominant NK cell population in secondary lymphoid organs and is particularly abundant in the decidua during pregnancy [26][27]. As PILRα-Ig bound to around 50% of the CD56^bright^ NK cells (Figure 1B), we sought to test whether the CD56^bright^ NK cells found in the decidua during pregnancy (dNK cells) would exhibit the same characteristics as the blood CD56^bright^ NK cells. We isolated dNK cells from first trimester decidua of healthy women who underwent elective termination of pregnancy and assessed the PILRα-Ig binding. As can be seen, most of the dNK cells, around 80%, bound PILRα-Ig (Figure 5A, quantified in 5B). Interestingly, other immune cells in the decidua which were CD56^negative^ (these are mostly T cells), also recognized by PILRα-Ig (Figure 5A).

Next, in order to assess IFNγ secretion and the direct killing capabilities of the dNK cells, we isolated PILRα-Ig expressing clones (which are negative for CD3, example is shown in Figure 5C). We observed that dNK cells incubated with PILRα expressing 721.221 targets secreted significantly more IFNγ (Figure 5D) and also demonstrated increased killing, compared to PILRα non-expressing targets (Figure 5E).

### NK cells express an unknown receptor against PILRα

**Figure 6:**
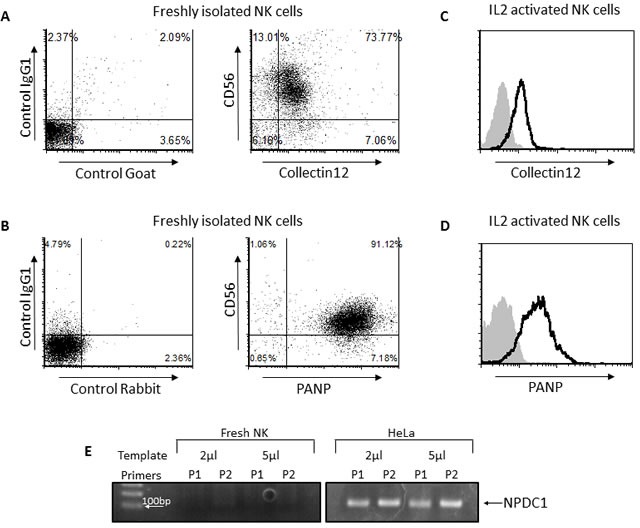
None of the known PILRα receptors are the PILRα receptor on NK cells **A.**, **B.** FACS dot blot staining of freshly isolated NK cells with PANP (A) and Collectin12 (B). The left dot plots in A and B are the control staining. **C.**, **D.** FACS staining of IL2 activated NK cells with PANP (C) and Collectin12 (D). Grey filled histograms are background control staining. Black line histograms represent specific staining, as indicated. **E.** PCR amplification of NPDC1 using two sets of specific exon spanning primers for NPDC1. cDNA derived from NK cells (left) or from HeLa cells (right) used as control. Figure show one representative experiment out of at least 3 performed.

PILRα has been shown to interact with three known proteins: PANP, Collectin12 and NPDC1 [17][18]. To test whether any of these proteins is the receptor for PILRα on NK cells, we stained freshly isolated NK cells with anti Collectin12 (Figure 6A) and anti-PANP (Figure 6B). As can be seen, all NK cells were stained with anti-Collectin12 and with anti-PANP mAbs (Figure 6A and 6B). Next, we stained IL2-activated NK cells with anti Collectin12 (Figure 6C) and anti-PANP (Figure 6D). Again, activated NK cells were stained with anti-Collectin12 and anti-PANP mAbs (Figure 6C and 6D). Since PILRα-Ig recognizes only a certain subset of NK cells (Figure 1A) and do not bind at all IL2 activated NK cells (Figure 1C), these results indicate that Collectin12 and PANP are not the PILRα receptor on NK cells.

To the best of our knowledge there are no commercial mAbs that recognize NPDC1. Thus, in order to detect the expression of this protein in NK cells we preformed PCR using two sets of specific exon spanning primers for NPDC1. According to our findings, NPDC1 was not expressed in NK cells, whereas it was expressed in HeLa cells, used as a positive control (Figure 6E). These combined results imply that none of the known PILRα receptors are the PILRα receptor on NK cells.

### PILRα-Ig interaction with its receptor on NK cells is protein-protein mediated and sialic acid independent

As none of the known PILRα receptors were identified as the designated PILRα receptor on NK cells, and since, as far as we know, there is no known NK cell activating receptor that demonstrates an expression pattern similar to the PILRα-Ig staining, we sought to evaluate the biochemical characteristic of the interaction between PILRα and its unknown NK receptor. We incubated freshly isolated NK cells with either Neuraminidase (NA) that catalyzes the hydrolysis of sialic acids from carbohydrates and glycoproteins or with Proteinase K, a serine protease.

**Figure 7:**
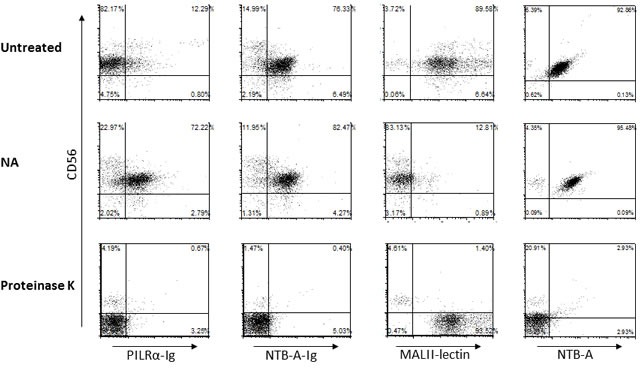
PILRα-Ig interaction with its receptor on NK cells is protein-protein mediated and not sialic acid dependent FACS staining of freshly isolated NK cells untreated (top), following NA treatment (center) or proteinase K treatment (bottom). NK cells were double stained with CD56 and PILRα-Ig, NTB-A-Ig (used as a control), MALII lectin and anti-NTB-A antibody. Figure show one representative experiment out of 3 performed.

In order to investigate the effect of the different treatments on the NK cell-PILRα interaction, we double stained the NK cells with CD56 and with PILRα-Ig, NTB-A-Ig or MALII lectin (Figure 7). NTB-A is a receptor expressed on NK cells, known to homophilically bind NTB-A and was therefore used as a positive control. Its expression on NK cells was monitored with a specific mAb (Figure 7, right), while NTB-A-Ig staining was used as an indicator for protein-protein interaction. Following NA treatment, NTB-A-Ig staining was slightly increased (Figure 7), indicating that sialic acids residues are not involved in the NTB-A homophilic interactions. Following Proteinase K treatment however, the binding of NTB-A-Ig and anti-NTB-A mAb to the treated cells was completely abolished (Figure 7). CD56 was abolished as well, since it is a proteinase K sensitive biomarker protein [28]. MALII is a lectin known to bind sialic acids, thus representing a positive control for the NA treatment. Indeed, following NA treatment the binding of MALII was abolished, whereas proteinase K treatment resulted in an altered staining of MALII (Figure 7).

Interestingly, while all PILRα receptors known to date were shown to bind PILRα in a sialic acid dependent manner [17][18], PILRα binding to its unknown NK cell receptor is protein-protein dependent and sialic acid independent. As can been seen, PILRα-Ig staining was completely eliminated following Proteinase K treatment, while NA treatment lead to an increase in PILRα-Ig staining (Figure 7, left), probably due to new non-specific epitopes that were revealed following NA treatment.

These results further demonstrate that the receptor for PILRα on NK cells is none of the known PILRα receptors and that in the PILRα binding to the unknown receptor on NK protein-protein interactions are involved.

## DISCUSSION

NK cells were discovered several decades ago [29]. Since then, several activating and many inhibitory receptors expressed on NK cells were reported. These receptors recognize numerous ligands expressed on target cells [9][30][31]. Nevertheless, the full spectrum of NK cell receptors and ligands that control NK cell activity is unknown. In particular, the identities of the ligands recognized by some of the activating NK cell receptors remains largely uncharacterized [2][4][30]. The identification of new NK cell receptors and ligands will contribute to the broad understanding of NK cell function and also potentially provide added value to establish new therapeutics in the field of cancer, autoimmunity and infectious diseases.

We started our research by seeking candidates that can potentially bind NKp44 and NKp46. PILRα was previously shown to bind O-glycosylated receptors [17][18], a characteristic which was inherent with the capacity of NKp46 to interact with its HA ligands [16]. Using fusion PILRα-Ig protein, we observed that PILRα-Ig binds NK cells, however, PILRα didn't bind NKp44 nor NKp46. Furthermore, PILRα-Ig exhibited a non-classical stochastic binding of freshly isolated NK cells and bound only to a portion of NK cells comprised mainly of the CD56^bright^ NK cell population.

Although the PILRα-binding receptor expressed on NK cells is unknown, we were able to distinguish between positive and negative clones by using PILRα-Ig. Positive clones showed specific and restricted activating features, such as IFNγ secretion and cytotoxicity when incubated with PILRα-expressing targets, establishing the role of PILRα as an activating ligand of NK cells. Interestingly, PILRα-Ig showed no binding to bulk IL2 activated NK cells while was bound to several isolated clones that were grown in the presence of IL2. This suggests that the designated PILRα receptor is not downregulated following IL2 activation, but is rather clonally restricted, an affect which could be undetectable in an activated bulk culture which is probably dominated by several fast growing NK clones.

Decidual NK cells comprise 50-70% of lymphocytes in the decidua during the first trimester of pregnancy and feature unique properties [26][27]. While the biological and clinical significance of their presence in early pregnancy is still under investigation, there are supporting evidences for both inhibitory and activating features, either in inhibition of anti-fetal immune response or in promotion of angiogenesis to support fetus implantation [26][32]. Recently it was shown that there was a significant enrichment of decidual-like NK cells within tumor infiltrating NK cell population which express pro-angiogenic factors and contribute to the tumor development [33]. Here we show that PILRα as a NK ligand, also activates dNK function by promoting killing and IFNγ secretion. This interaction of PILRα with NK cells in the decidua could be functionally relevant since other cells that are present in the decidua express PILRα [19][20].

In search for the receptor of PILRα expressed by NK cells, we tested the known PILRα receptors: PANP, Collectin12 and NPDC1. In mice models, mPILRα was shown to bind mCD99 on NK cells, however, such an interaction was not observed in human NK cells and all NK cells express CD99, both prior to and following IL2 activation [17][18]. According to our results, none of the known PILRα receptors seems to be the PILRα receptor on NK cells. Furthermore, we demonstrated that sialic-acid independent protein-protein dependent interactions are involved in the PILRα interaction with NK cells, a fact that further exclude the currently known receptors of PILRα.

PILRα is expressed primarily on myeloid cells, including granulocytes, monocytes, macrophages and dendritic cells [19][20]. Curiously, PILRα contains two ITIM motifs that trigger an inhibitory signaling cascade in myeloid cells [34], while exhibiting activating features as a ligand for NK cells. Similarly, PVR (CD155) functions as a ligand for NK cells by binding an inhibitory receptor TIGIT and two co-stimulating receptors DNAM1 and CD96 [35], while it inhibits dendritic cell function via its ITIM motif [36].

In summary, we discovered a novel and unique activating ligand for NK cells named PILRα. The identity of its receptor is unknown and we hope to discover it in the future.

## MATERIALS AND METHODS

### Primary cells and cell culture

Peripheral blood lymphocytes (PBLs) were isolated from healthy donors using Ficoll gradient separation (Lymphoprep™, Stemcell). NK cells were isolated from the PBLs using an NK negative selection kit (EasySep™, Stemcell). CD56bright isolation was performed using CD56+CD16- isolation kit (MACS, Miltenyi Biotec). Isolation of decidual lymphocytes was performed as previously described [37]. The cell lines used in this paper included 293T, BW and 721.221 cells. 293T cells were maintained in Dulbecco's modified Eagle's medium, BW and 721.221 cells were maintained in RPMI medium, both supplemented with 10% fetal calf serum. All cells were incubated in a humidified atmosphere of 5% CO2 and 95% air at 37°C.

### Generation of fusion proteins

Sequences encoding PILRα, CLEC5A (control-Ig) and NTB-A were amplified by PCR using the following primers: PILRα-Ig for 5′ - GGG AATTCGCCGCCACCATGGGTCGGCCCCTGCTG -3′, rev 5′-GGGGATCCGCAGTCTCCAGACTTATGTGCC -3′; CLEC5A Ig for 5′-GGACTAGTCCAC AGATTTTTAACAAAAGTA -3′, rev 5′-GGGCTAGCTCATTTGGCATTCTTCTCACAGAT -3′; NTB-A-Ig for CCCACCGG TGCCGCCACCATGTTGTGGCTGTTCCAATCG-3′, rev 5′-GGGACTCATTTT GGTATCTGTATATTG-3′. These PCR-generated fragments were cloned into the mammalian expression vector containing the Fc portion of human IgG1 (mutated to abolish the Fc receptor binding site), generated in 293T cells and Ig-fusion proteins were purified on a protein G column as described [38]. Sequencing of the constructs revealed that cDNA of all Ig-fusion proteins was in frame with the human Fc genomic DNA and were identical to the reported sequences. All Ig-fusion proteins used in this work migrate as a single band on standard non-reduced SDS-PAGE gels and each was regularly assayed by SDS-PAGE to ensure the proteins had not degraded. Protein purity of all Ig fusion proteins used in this study was around 100%.

### Antibodies and flow cytometry

NK cells were defined by being positive for anti-CD56-PE (BD Biosciences) and negative for CD3 staining. FACS staining was performed using conjugated antibodies against PILRα (R&D systems), NKp44 (BioLegend) and NKp46 (R&D systems). Binding of antibodies against PANP (Abgent), Collectin12 (R&D systems) and NTB-A (BioLegend) were detected using the compatible secondary antibodies; Alexa Fluor 647-conjugated AffiniPure donkey anti-rabbit IgG, Alexa Fluor 647-conjugated AffiniPure donkey anti-goat IgG and Alexa Fluor 647-conjugated AffiniPure goat anti-mouse IgG (all purchased from Jackson ImmunoResearch). The anti-HA1 mAb (H17-L2) was a kind gifts from Jonathan Yewdell, National Institutes of Health. Biotinylated-MALII (vector laboratories) was detected using streptavidin APC antibody (BioLegend). For the fusion proteins detection, a secondary antibody staining of anti-human APC (Jackson ImmunoResearch) was used. All staining were analyzed by flow cytometry using the CellQuest software.

### BW assay

The generation of chimeric NKp44 and NKp46 and expression in BW cells was previously described [21]. The BW assay was performed as previously described [21]. Briefly, BW or BW transfected cells were incubated with irradiated targets (6000rad) at 1:1 effector to target (E:T) ratio. After 48h, the supernatants were collected and the level of interleukin-2 (IL2) was quantified by sandwich ELISA using anti IL2 mAbs (BioLegend).

### IFNγ secretion assay

Freshly isolated NK cells or decidual NK cells (50,000 cells/well) were incubated either alone or with 721.221 target cells (50,000 cells/well) for 5 hours in 37°C, in a 96 well U-shaped bottomed plate. Following incubation, the supernatants were analyzed for the presence of IFNγ. The ELISA assays for the detection of human IFNγ (BioLegend) were performed in accordance with the manufacturer's protocol and reagents.

### NK cell degranulation assay

Analysis of cell surface–mobilized CD107a (degranulation assay) has been previously described [39]. Briefly, NK cells (50,000) were incubated either alone or with 721.221 target cells (50,000 cells), APC conjugated CD107a (BioLegend) and PE conjugated CD56 (BioLegend), for 2 hours in 37°C. Following incubation, CD107a expression was analyzed using flow cytometry.

### NK cell cytotoxicity assay

NK Target cells were grown over night in the presence of ^35^S added to a Methionine-free media (Sigma). Prior to incubation with the effectors, target cells were washed, counted, and 5000 cells/well were plated. For each target, the spontaneous ^35^S release was calculated from cells which were not incubated with effector cells, and maximum ^35^S release was calculated by adding 0.1M of NaOH to the target cells. The level of ^35^S release was measured after 5 hours of incubation with effectors by a MicroBeta Plate Counter (Perkin Elmer).

### Biochemical characterization

Freshly isolated NK cells (1,000,000) were incubated in 37°C with 5μl of either Neuraminidase (Millipore) or Proteinase K (Sigma). Following 30 minutes incubation, the cells were washed and divided equally for the appropriate FACS staining.
